# Changes in the Small RNA Expression in Endothelial Cells in Response to Inflammatory Stimulation

**DOI:** 10.1155/2021/8845520

**Published:** 2021-04-27

**Authors:** Peixi Liu, Liuxun Hu, Yuan Shi, Yingjun Liu, Guo Yu, Yingjie Zhou, Qingzhu An, Wei Zhu

**Affiliations:** ^1^Department of Neurosurgery, Huashan Hospital of Fudan University, Shanghai 200040, China; ^2^Neurosurgical Institute of Fudan University, Shanghai 200040, China; ^3^Shanghai Clinical Medical Center of Neurosurgery, Shanghai 200040, China; ^4^Shanghai Key Laboratory of Brain Function and Restoration and Neural Regeneration, Shanghai 200040, China; ^5^Department of Hand surgery, Huashan Hospital of Fudan University, Shanghai 200040, China

## Abstract

**Objective:**

Endothelial cell inflammation is a common pathophysiological process in many cardiovascular and cerebrovascular diseases. Small RNA is a kind of short nonprotein coding RNA molecule. Changes in the small RNA expression in endothelial cells have been linked to the development of cardiovascular and cerebrovascular diseases. We investigated and verified differentially expressed small RNAs in endothelial cells in response to inflammatory stimulation.

**Methods:**

Primary rat endothelial cells were obtained from Sprague-Dawley rats and treated with 10 ng/ml TNF-*α* for 24 hours. Small RNA sequencing was used to generate extensive small RNA data. Significantly differentially expressed small RNAs identified in the analysis were further confirmed by quantitative reverse transcription polymerase chain reaction (qRT-PCR). Then, we investigated the tissue-specific small RNA expression after RNA extraction from different tissues.

**Results:**

Small RNA sequencing demonstrated that 17 miRNAs, 1 piRNA, 10 snoRNAs, and 7 snRNAs were significantly differentially expressed. qRT-PCR identified 3 miRNAs, 2 snoRNAs, and 2 snRNAs with significantly different expression. Analysis of the tissue-specific expression showed that rno-miR-126a-5p was predominantly expressed in the lung, rno-miR-146a-5p in the intestines, and rno-novel-178 in the heart. Rno-piR-017330 was mainly expressed in the muscle. snoR-8966.1 was predominantly expressed in the bone. snoR-6253.1 was mostly expressed in the vessels and bone. snR-29469.1 was mainly expressed in the bone, and snR-85806.1 was predominantly expressed in the vessels and bone.

**Conclusions:**

We report for the first time the expression of small RNAs in endothelial cells under inflammatory conditions. TNF-*α* can regulate the expression of small RNAs in endothelial cells, and their expression is tissue-specific.

## 1. Introduction

Small RNAs are key regulators of biological activities and play an important role in the regulation of the gene expression, biological ontogenesis, metabolism, and the occurrence of diseases and other physiological processes. Generally, small RNAs include microRNAs (miRNAs), small noncoding RNAs (ncRNAs), small interfering RNAs (siRNAs), small nucleolar RNAs (snoRNAs), P-element-induced wimpy testis- (PIWI-) interacting RNAs (piRNAs), and repeat-associated siRNAs (rasiRNAs). Small RNAs regulate the growth and development of organisms and the occurrence of disease through a variety of pathways, including mRNA degradation, translational inhibition, heterochromatin formation, and DNA removal.

Endothelial cell inflammation is a common process that is closely related to the pathophysiological mechanisms of the cardiovascular and cerebrovascular systems. Although several specific changes in miRNAs and other small RNAs in inflamed endothelial cells have been elucidated, a comprehensive analysis of small RNA changes has not been performed.

In this study, primary rat endothelial cells were isolated and subjected to inflammatory stimuli. To gain insights into small RNA changes in endothelial cells in response to inflammation, we used small RNA sequencing (small RNA-seq) to generate extensive small RNA data, along with RNA-seq data. We identified differentially expressed small RNAs and performed tissue-specific analysis. These findings might provide novel insight into disease pathogenesis and early diagnosis.

## 2. Materials and Methods

### 2.1. Endothelial Cell Isolation and Culture

The animal protocol was approved by the Institutional Animal Care and Use Committee (IACUC), and the experimental protocol was reviewed and approved by the Ethics Committee of Fudan University, Shanghai, China. Adult male Sprague-Dawley rats (Shanghai SLAC Laboratory Animal Co., Ltd., Shanghai, China) weighing 200 to 250 g were used. The animals were anesthetized with Forane (Abbott, Shanghai, China) using a vaporizer (Matrx, Midmark, Dayton, OH). Vessel segments were obtained from a healthy rat abdominal aorta. Samples were turned inside out to expose the endothelium. The ends of the blood vessels were ligated, and the samples were collected in DMEM containing 5% penicillin/streptomycin. Tissue segments were washed 3 times with phosphate-buffered saline (PBS) supplemented with 1% penicillin/streptomycin. A pancreatin-trypsin solution was used to digest the tissue for 3 minutes. DMEM (Gibco, Grand Island, NY, USA) containing 10% FBS (Gibco) and 1% penicillin/streptomycin was used to terminate the digestion. Cells were cultured on fibronectin-coated dishes with endothelial cell medium (ECM, ScienCell, San Diego, CA) at 37°C in a 5% CO_2_ incubator. The medium was changed every 3 days. TNF-*α* stimulation was used to mimic an inflammatory environment. The cells in the TNF-*α* group were treated with 10 ng/ml TNF-*α* for 24 hours.

### 2.2. Small RNA Sequencing (RNA-seq)

The quality of the original sequencing data was evaluated by FastQC using cutadapt to remove the adaptor and trimmomatic to remove low-quality bases at both ends and read filtering. BLASTn was used to compare reads with the small RNA, transfer RNA, small nuclear RNA (snRNA), and snoRNA sequences in the Rfam database, and the number and percentage of mapped reads were calculated. Bowtie was used to compare the read sequences with the exon and intron sequences of the species, and the number and percentage of mapped reads were calculated. Reads that mapped to an exon in the alignment but did not map to an intron were filtered out, after which bowtie was used to align the reads with the reference genome sequence, the number and percentage of mapped reads were calculated, and the reads that did not align were filtered out.

### 2.3. Quantitative Reverse Transcription Polymerase Chain Reaction (qRT-PCR)

Total RNA was isolated from cells with TRIzol Reagent (Invitrogen) according to the manufacturer's instructions. After the reverse transcription reaction, RT-PCR was performed with the ABI 7900HT system using SYBR Premix (Takara, Dalian, China) according to the manufacturer's instructions. The RT-PCR conditions were as follows: denaturation at 95°C for 10 s, followed by 40 cycles at 95°C for 10 s, and 60°C for 30 s. A dissociation step was added at the end of the amplification procedure. No nonspecific amplification was observed based on the dissociation curve. Glyceraldehyde 3-phosphate dehydrogenase (GAPDH) was used as an internal control. The data were analyzed using the comparative Ct (2^−*ΔΔ*Ct^) method and expressed as the fold change relative to the respective control. Each sample was analyzed in triplicate. The primer sequences used in this study are shown in Table 1.

### 2.4. Statistical Analysis

The statistical analysis was performed using IBM SPSS Statistics, and graphs were generated by GraphPad Prism. Independent sample *t*-tests were used to analyze the qPCR mRNA expression levels. *P* values less than 0.05 were considered significant.

## 3. Results

### 3.1. Identification of Differentially Expressed miRNAs and Tissue-Specific Analysis

**Figure 1:**
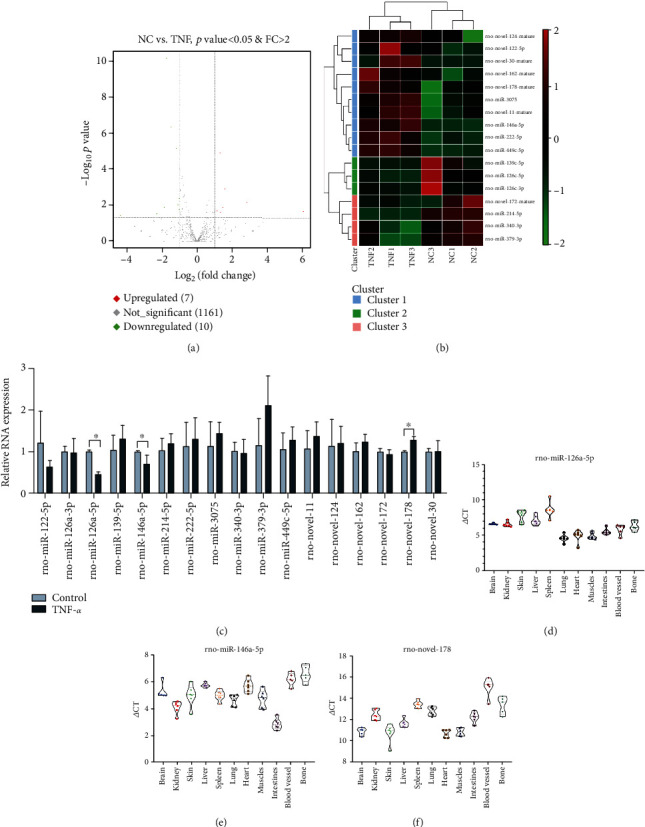
Analysis of the expression of miRNAs in endothelial cells and rat tissue. (a) A volcano plot was used to show the differences in miRNA expression levels between the TNF-*α* group and NC group. (b) Heatmap analysis of the differences in miRNA expression levels. (c) The miRNA expression in endothelial cells stimulated with TNF-*α* was detected by qRT-PCR. (d) The Rno-miR-126a-5p expression in the rat tissue was detected by qRT-PCR. (e) The Rno-miR-146a-5p expression in the rat tissue was detected by qRT-PCR. (f) The novel-178 expression in the rat tissue was detected by qRT-PCR. Data are presented as the mean ± S.D. ^∗^ indicates *P* < 0.05.

miRNAs play an important role in cellular processes; so, we performed small RNA-seq to analyze the miRNA expression in endothelial cells stimulated with TNF-*α*. The small RNA-seq results showed that ten miRNAs were upregulated, and seven miRNAs were downregulated in endothelial cells stimulated with TNF-*α* (Figures 1(a) and 1(b)). Then, we detected the expression of miRNAs in endothelial cells treated with TNF-*α* using qRT-PCR. The results showed that the expression of rno-miR-126a-5p and rno-miR-146a-5p was higher in the TNF-*α* group than in the control group. However, the expression of rno-novel-178 was lower in the TNF-*α* group than in the control group (Figure 1(c)). We also detected the expression of rno-miR-126a-5p, rno-miR-146a-5p, and rno-novel-178 in different rat tissues (Figures 1(d)–1(f)).

### 3.2. Identification of Differentially Expressed piRNAs and Tissue-Specific Analysis

**Figure 2:**
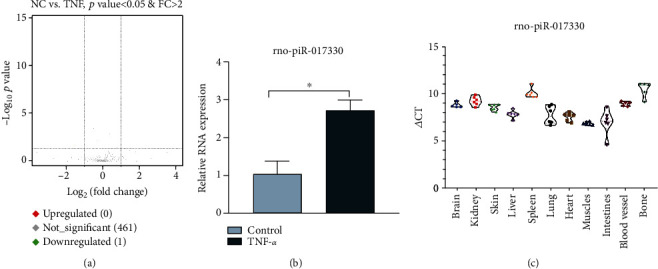
Analysis of the piRNA expression in endothelial cells and rat tissue. (a) A volcano plot was used to show the difference in piRNA expression levels between the TNF-*α* group and NC group. (b) The rno-piR-017330 expression in endothelial cells stimulated with TNF-*α* was detected by qRT-PCR. (c) The rno-piR-017330 expression in the rat tissue was detected by qRT-PCR. Data are presented as the mean ± S.D. ^∗^ indicates *P* < 0.05.

PiRNA, a type of small RNA with a length of approximately 30 nt, is expressed in mammalian germ cells and plays its regulatory role only when associated with members of the PIWI protein family. The small RNA-seq results showed that one piRNA was upregulated in endothelial cells stimulated with TNF-*α* (Figure 2(a)). Then, we detected the piRNA expression in endothelial cells treated with TNF-*α* using qRT-PCR. The results showed that the expression of rno-piR-017330 was higher in the TNF-*α* group than in the control group. However, the expression of rno-novel-178 was lower in the TNF-*α* group than in the control group (Figure 2(b)). We also detected the expression of rno-piR-017330 in different rat tissues (Figure 2(c)).

### 3.3. Identification of Differentially Expressed snoRNA and Tissue-Specific Analysis

**Figure 3:**
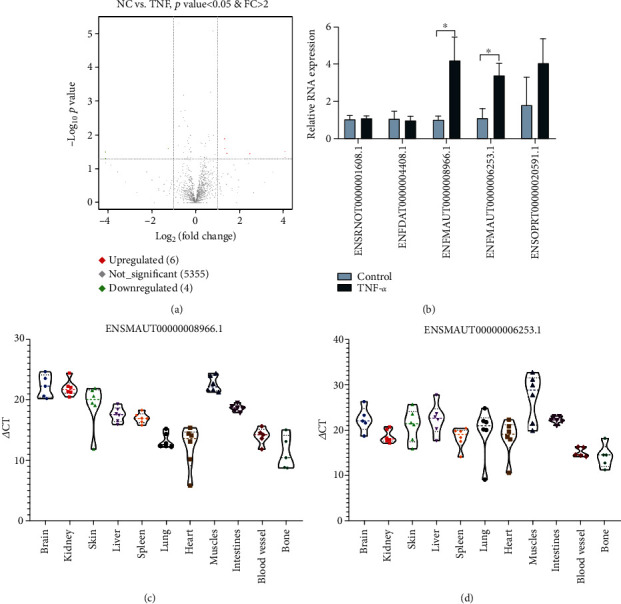
Analysis of the expression of snoRNAs in endothelial cells and rat tissue. (a) A volcano plot was used to show the differences in snoRNA expression levels between the TNF-*α* group and NC group. (b) The snoRNA expression in endothelial cells stimulated with TNF-*α* was detected by qRT-PCR. (c) The ENSMAUT00000008966.1 expression in the rat tissue was detected by qRT-PCR. (d) The ENSMAUT00000006253.1 expression in the rat tissue was detected by qRT-PCR. Data are presented as the mean ± S.D. ^∗^ indicates *P* < 0.05.

snoRNA has recently become a hot spot in biological research. This small noncoding RNA, which has conserved structural elements, is encoded by introns and is distributed in the nucleolus of eukaryotic cells. The small RNA-seq results showed that the expression of snoRNAs in endothelial cells was regulated by TNF-*α* stimulation (Figure 3(a)). Then, we detected the snoRNA expression in endothelial cells treated with TNF-*α* using qRT-PCR. The results showed that the expression of snoRNAs ENSMAUT00000008966.1 and ENSMAUT00000006253.1 was higher in the TNF-*α* group than in the control group (Figure 3(b)). We also detected the expression of snoRNA ENSMAUT00000008966.1 and ENSMAUT00000006253.1 in different rat tissues (Figures 3(c) and 3(d)).

### 3.4. Identification of Differentially Expressed snRNAs and Tissue-Specific Analysis

**Figure 4:**
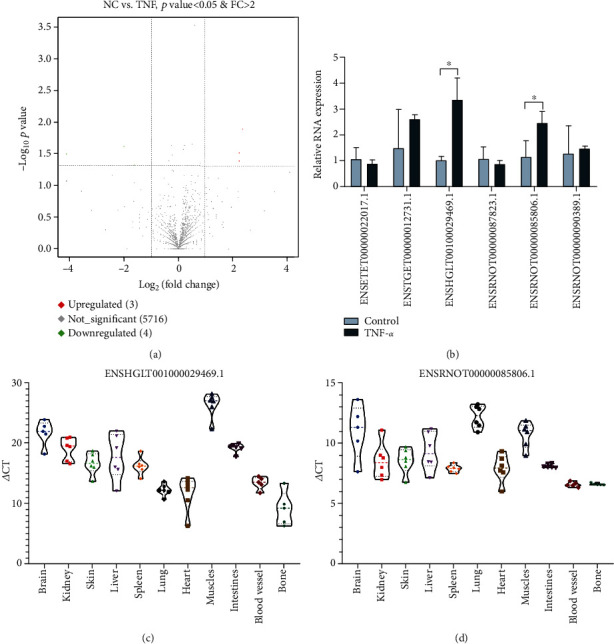
Analysis of the expression of snRNAs in endothelial cells and rat tissue. (a) A volcano plot was used to show the difference in snRNA expression levels between the TNF-*α* group and NC group. (b) The snoRNA expression in endothelial cells stimulated with TNF-*α* was detected by qRT-PCR. (c) The ENSHGLT00100029469.1 expression in the rat tissue was detected by qRT-PCR. (d) The ENSRNOT00000085806.1 expression in the rat tissue was detected by qRT-PCR. Data are presented as the mean ± S.D. ^∗^ indicates *P* < 0.05.

snRNA is the main component of the RNA spliceosome in posttranscriptional processing in eukaryotes and participates in the processing of mRNA precursors. The small RNA-seq results showed that the expression of snRNAs in endothelial cells was regulated by TNF-*α* stimulation (Figure 4(a)). Then, we detected the snoRNA expression in endothelial cells treated with TNF-*α* using qRT-PCR. The results showed that the expression of snRNA ENSHGLT00100029469.1 and ENSRNOT00000085806.1 was higher in the TNF-*α* group than in the control group (Figure 4(b)). We also detected the expression of snoRNAs ENSHGLT00100029469.1 and ENSRNOT00000085806.1 in different rat tissues (Figures 4(c) and 4(d)).

## 4. Discussion

In previous studies, vascular inflammation was related to the occurrence and development of atherosclerosis, ischemic stroke, cerebral aneurysm, rheumatoid arthritis, and chronic venous disease [1–4]. It is also related to treatment decisions, prognosis, and risk of future cardiovascular and cerebrovascular events. In this study, we employed TNF-*α* to mimic this pathophysiological process. TNF-*α* induced cell inflammation via various mechanisms [5–7]. And it is also used as a mature cellular modeling technique.

Endothelial cells have various important functions, including maintenance of vascular haemostasis and secretion of several vasoactive and antithrombogenic mediators. Intravascular blood flow wall shear stress, cyclic stretch, oxidative stress, dyslipidaemia, mechanical injury, and other factors are related to pathological changes in endothelial cells. The interaction between different types of vascular wall cells is also important for endothelial cell inflammation [8, 9]. We extracted arterial endothelial cells from the aorta. Abdominal aorta is typical vascular tissue, and it involves endothelial cells and smooth muscle cells. During tissue specific analysis, the obvious small RNAs changes might be disturbed.

Small RNAs, a large class of regulatory molecules that exists in almost all organisms, include miRNAs, ncRNAs, siRNAs, snoRNAs, piRNAs, and rasiRNAs,. Small RNAs regulate the growth and development of organisms and the occurrence of diseases through a variety of mechanisms, including mRNA degradation, translational inhibition, heterochromatin formation, and DNA removal. Small RNA transcriptome sequencing is a new method and powerful tool for the identification and quantitative analysis of small RNAs.

miRNAs are small noncoding single-stranded RNAs 18-25 nucleotides in length. A mature miRNA functions by complementary pairing with the 3′-noncoding region (3′-UTR) of its target gene. Full complementarity with the 3′-UTR of the target gene leads to accelerated degradation of the target mRNA, while partial complementarity inhibits target protein synthesis. As a negative regulator of the gene expression, miRNA can affect many important human physiological and pathological processes, including differentiation, proliferation, apoptosis, migration, homeostasis, and various disease processes. A number of miRNAs can regulate the physiological functions of vascular endothelial cells, and miRNAs related to vascular endothelial cell inflammation can become new targets for the treatment of cardiovascular and cerebrovascular diseases. In our current study, we found that the expression of rno-miR-126a-5p and rno-miR-146a-5p decreased under TNF-*α* stimulation conditions, while the expression of novel-178 increased under TNF-*α* stimulation conditions. Studies have reported that miR-126a-5p reduces the occurrence of abdominal aortic aneurysm by inhibiting the expression of ADAMTS-4 [10]. Many studies have investigated the relationship between miR-146a-5p and the TNF-*α* signaling pathway [11–13]. Rno-novel-178, a novel miRNA that we identified in this study, may function as a proinflammatory miRNA. Its relationship with TNF-*α* and its role in endothelial cells need further verification.

PiRNA is a type of small RNA with a length of approximately 30 nt and expressed in mammalian germ cells that plays its regulatory role only when associated with members of the PIWI protein family. At present, an increasing number of studies have shown that the regulation of the growth and development of germ cells by piRNAs is related to gene silencing caused by the Piwi-piRNA complex, but because research on piRNAs is still nascent, some of the specific functions of this complex are still under research. There are no reports on the relationship between piRNAs and TNF-*α*, and the role of piRNAs in endothelial cells is unknown. Previous studies reported that piRNAs exist only in germ cells; however, we found that piRNAs are also present in endothelial cells, although their specific functions need to be further elucidated.

snoRNA is a type of small noncoding RNA widely distributed in the nucleolus of eukaryotic cells. It has conserved structural elements and is divided into three categories: box C/D snoRNA, box H/ACA snoRNA, and MRP RNA. Among them, box C/D and box H/ACA are the main types of known snoRNAs, which guide the methylation and pseudouridylation modification of ribosomal RNA by base pairing, respectively. Studies have found that in addition to playing a role in ribosomal RNA biosynthesis, snoRNAs can guide the posttranscriptional modification of snRNA, tRNA, and mRNA. In addition, there are a considerable number of snoRNAs with unknown functions, which are called orphan snoRNAs. Among mammalian orphan snoRNAs, imprinted snoRNA (imprinted snoRNA) is the most unique group, which is encoded by the genomic imprinted region and has obvious tissue expression specificity. The identification of snoRNA-like members in prokaryotic archaea indicates the ancient origin of these noncoding RNA family members, and the existence of a large number of snoRNA transposons in mammals provides a new way for people to explore the amplification and functional evolution of snoRNAs in the genome. We first studied the relationship between snoRNAs and TNF-*α* in endothelial cells. A number of snoRNAs were observed to be expressed in endothelial cells. In addition, we found that the expression of ENSMAUT00000006253.1 in tissues is vascular-specific; so, the role of snoRNAs in endothelial cells needs to be further explored.

snRNA is the main component of the RNA spliceosome in the posttranscriptional processing of eukaryotes and participates in the processing of mRNA precursors. In mammals, its length is approximately 100-215 nucleotides, and it is divided into 7 categories, numbered U1 ~ U7 based on the number of U residues. SnRNA exists only in the cell nucleus. Usually, snRNA does not exist freely but interacts with protein to form a complex called small nuclear ribonucleoprotein particle (snRNP). snRNA does not participate in protein synthesis activities but plays an important role in RNA processing. U3 snRNA is related to the maturation of 28S rRNA in the nucleolus, while U1 is related to the splicing and processing of precursor mRNA in the nucleus. The protein component of snRNA has nuclease and ligase activity, which can cut the transcript at the intron-exon junction and connect the two free ends. However, the role of snRNA in endothelial cells and its relationship with the TNF-*α* signaling pathway have not yet been reported. Similarly, we found that the expression of snRNA ENSRNOT00000085806.1 has vascular tissue specificity. The function and potential target of ENSRNOT00000085806.1 in endothelial cells will be explored and verified in our subsequent studies.

Oxidative stress plays an important role in stroke, and endothelium inflammation is inevitable. Oxidative stress is often a trigger for inflammation. Increasing evidence demonstrates that oxidative stress responses participate in the pathophysiological processes of secondary brain injury following intracerebral haemorrhage, cerebral ischemia, and subarachnoid hemorrhage [14–17]. Small RNAs are opportunities to be potential targets in antioxidation and anti-inflammation therapy. Unfortunately, few studies have focused on small RNA changes in endothelial cells under inflammatory conditions. Anti-inflammatory therapy is a frequently discussed clinical issue that includes new drug research, drug-target protein detection, disease mechanism exploration, vascular disease radiological diagnosis, and intravascular research [18, 19]. Through screening, we will verify the target genes of above small RNAs in the subsequent in vivo and in vitro studies. Exploration of endothelial cells in response to inflammatory stimulation may shed light on this field.

## 5. Conclusions

To our knowledge, no studies have reported the expression of small RNA in endothelial cells under TNF-*α* stimulation. We found that TNF-*α* can regulate the expression of small RNAs in endothelial cells, and our study adds new information to better understand this phenomenon. This finding may shed light on the diagnosis and treatment of endothelial cell inflammation-related diseases. However, the function of small RNAs in endothelial cells needs further study.

## Figures and Tables

**Table 1:** Sequence of primers used for qRT-PCR.

Gene		Sequence (5′ to 3′)
rno-miR-122-5p		GCAGTGGAGTGTGACAATG
rno-miR-126a-3p		GCAGTCGTACCGTGAGT
rno-miR-126a-5p		CGCAGCATTATTACTTTTGGT
rno-miR-139-5p		GTCTACAGTGCACGTGTC
rno-miR-146a-5p		GCAGTGAGAACTGAATTCCA
rno-miR-214-5p		CGCAGAGAGTTGTCATGTG
rno-miR-222-5p		GGGCTCAGTAGCCAGT
rno-miR-3075		GTCTGGGAGCAGCCA
rno-miR-340-3p		GCAGTCCGTCTCAGTTAC
rno-miR-379-3p		CGCAGCTATGTAACATGGTC
rno-miR-449c-5p		GAGGCAGTGCATTGCT
rno-novel-11-mature		AGTAGGGTCTGTTCTGTGTC
rno-novel-124-mature		CAGTTCTTCTGAAAGCTTCTGA
rno-novel-162-mature		GGTGGGACCTGTGGT
rno-novel-172-mature		GCAGCTGGGCTACACA
rno-novel-178-mature		CGCAGTTGGTACCTGTTTC
rno-novel-30-mature		CAGGTCCACTCTGCTGA
rno_piR_017330	F:	GCTGATCAACTGCCTGAC
ENSMAUT00000008966.1	F:	TGATTCCTGTTGCTTTGCCTG
	R:	GTCTTTCAGAAGAGGTAGTGACTGA
ENSMAUT00000006253.1	F:	AGGTGCTTGAGTTGTTGACCT
	R:	ACACCAGTGGAGTCCTGACA
ENSOPRT00000020591.1	F:	GCTGTGCGTGATGACA
	R:	CAGGCAGTTTCCTCAGG
ENSFDAT00000004408.1	F:	TGGCCAAAGATGAGAACTCTAAC
	R:	GCCTCAGGTAAATCCTTTAACCC
ENSRNOT00000091608.1	F:	ACTGGTCTGCAGCTGTCTTT
	R:	TGTCTGTCACGCATATTCCTCT
ENSRNOT00000090389.1	F:	AGCTTTGTGCAGTGGCAGTA
	R:	ATATTGCAAGTTGTCATGGCG
ENSVPAT00000017203.1	F:	AGCCAATGGGGTTTATCCGA
	R:	TTAAGTTGGAACATGGAAACTGG
ENSRNOT00000085806.1	F:	GCTTTGAGTCCCTGAGGACA
	R:	GATGGGCCAATTCAAAGCCC
ENSRNOT00000087823.1	F:	TCGGCCTTTTGGCTAAGATCA
	R:	TGGGCAAGAGGAGAGGTTGA
ENSHGLT00100029469.1	F:	AGCAGCACGTACATATTAGAGCA
	R:	GAGTTTGTATGTCAGCCTTGTGC
ENSTGET00000012731.1	F:	CTGCTCACTTCATCAGCACA
	R:	ACGTATTTGTGCATCAGGGGT
ENSETET00000022017.1	F:	AGGGCGAGGCTTATCCATTG
	R:	GCAGTTCCCCACTACCACAA
U6 F		GCAGCGTGAAGCGTTC
RT primer		CAGGTCCAGTTTTTTTTTTTTTTTVN
rno GAPDH	F:	GGACCAGGTTGTCTCCTGTG
	R:	CATTGAGAGCAATGCCAGCC

## Data Availability

The data used to support the findings of this study are available from the corresponding author upon request.

## Supplementary Materials

Supplementary Materials Supplementary materials include raw data of sequencing for microRNA and other small RNAs (piRNA, snRNA, and snoRNA).
