# Supplementary material for: Changes in the Small RNA Expression in Endothelial Cells in Response to Inflammatory Stimulation
Source: Oxid Med Cell Longev. 2021 Apr 27;2021:8845520. doi: 10.1155/2021/8845520 (PMC8133845; doi:10.1155/2021/8845520)
Supplement: Supplementary Materials — include raw data of sequencing for microRNA and other small RNAs (piRNA, snRNA, and snoRNA). [file 8845520.f1.docx]

We have a raw data file of RNA sequencing as a supplementary material to upload, about 4GB. But the network of the submission system has a bit of a lag and we are unable to upload this file in the submission system.

When we contacted the Hindawi staff via email, he said that uploading could be done via Baidu Cloud Drive, so we uploaded the raw data to the disk. We are very sorry for the inconvenience.

link：https://pan.baidu.com/s/1VxcZq7pSLfQSvWS8chp6PQ

password：6viq
